# Evaluating a Remote Monitoring Program for Respiratory Diseases: Prospective Observational Study

**DOI:** 10.2196/51507

**Published:** 2023-11-24

**Authors:** Malik A Althobiani, Yatharth Ranjan, Joseph Jacob, Michele Orini, Richard James Butler Dobson, Joanna C Porter, John R Hurst, Amos A Folarin

**Affiliations:** 1 Respiratory Medicine University College London London United Kingdom; 2 Interstitial Lung Disease Service University College London Hospital London United Kingdom; 3 Department of Respiratory Therapy Faculty of Medical Rehabilitation Sciences King Abdulaziz University Jeddah Saudi Arabia; 4 Department of Biostatistics and Health Informatics Institute of Psychiatry, Psychology and Neuroscience King’s College London London United Kingdom; 5 Satsuma Lab Centre for Medical Image Computing University College London London United Kingdom; 6 Institute of Cardiovascular Science University College London London United Kingdom; 7 National Institute for Health and Care Research, Biomedical Research Centre at South London and Maudsley NHS Foundation Trust and King’s College London London United Kingdom; 8 Institute of Health Informatics University College London London United Kingdom; 9 National Institute for Health and Care Research, Biomedical Research Centre at University College London Hospitals National Institute for Health Foundation Trust London United Kingdom

**Keywords:** remote monitoring, home health care, mHealth, mobile health, apps, applications, wearables, passive data collection, data collection, retention, engagement, attrition, dropout, spirometry, oximetry, home based, machine learning, artificial intelligence, chronic obstructive pulmonary disease, COPD, pulmonary, lungs, respiratory, interstitial lung disease, ILD, COVID-19, respiratory diseases, lung, chronic, SARS-CoV-2, monitoring, observational, cohort, feasibility, usability, acceptability, community based, self-management

## Abstract

**Background:**

Patients with chronic respiratory diseases and those in the postdischarge period following hospitalization because of COVID-19 are particularly vulnerable, and little is known about the changes in their symptoms and physiological parameters. Continuous remote monitoring of physiological parameters and symptom changes offers the potential for timely intervention, improved patient outcomes, and reduced health care costs.

**Objective:**

This study investigated whether a real-time multimodal program using commercially available wearable technology, home-based Bluetooth-enabled spirometers, finger pulse oximeters, and smartphone apps is feasible and acceptable for patients with chronic respiratory diseases, as well as the value of low-burden, long-term passive data collection.

**Methods:**

In a 3-arm prospective observational cohort feasibility study, we recruited 60 patients from the Royal Free Hospital and University College Hospital. These patients had been diagnosed with interstitial lung disease, chronic obstructive pulmonary disease, or post–COVID-19 condition (n=20 per group) and were followed for 180 days. This study used a comprehensive remote monitoring system designed to provide real-time and relevant data for both patients and clinicians. Data were collected using REDCap (Research Electronic Data Capture; Vanderbilt University) periodic surveys, Remote Assessment of Disease and Relapses–base active app questionnaires, wearables, finger pulse oximeters, smartphone apps, and Bluetooth home-based spirometry. The feasibility of remote monitoring was measured through adherence to the protocol, engagement during the follow-up period, retention rate, acceptability, and data integrity.

**Results:**

Lowest-burden passive data collection methods, via wearables, demonstrated superior adherence, engagement, and retention compared with active data collection methods, with an average wearable use of 18.66 (SD 4.69) hours daily (77.8% of the day), 123.91 (SD 33.73) hours weekly (72.6% of the week), and 463.82 (SD 156.70) hours monthly (64.4% of the month). Highest-burden spirometry tasks and high-burden active app tasks had the lowest adherence, engagement, and retention, followed by low-burden questionnaires. Spirometry and active questionnaires had the lowest retention at 0.5 survival probability, indicating that they were the most burdensome. Adherence to and quality of home spirometry were analyzed; of the 7200 sessions requested, 4248 (59%) were performed. Of these, 90.3% (3836/4248) were of acceptable quality according to American Thoracic Society grading. Inclusion of protocol holidays improved retention measures. The technologies used were generally well received.

**Conclusions:**

Our findings provide evidence supporting the feasibility and acceptability of remote monitoring for capturing both subjective and objective data from various sources for respiratory diseases. The high engagement level observed with passively collected data suggests the potential of wearables for long-term, user-friendly remote monitoring in respiratory disease management. The unique piloting of certain features such as protocol holidays, alert notifications for missing data, and flexible support from the study team provides a reference for future studies in this field.

**International Registered Report Identifier (IRRID):**

RR2-10.2196/28873

## Introduction

### Background

To gain deeper insights into the etiology of respiratory diseases and identify personalized treatment plans for effective management, research on diverse populations is needed to continuously assess physiological parameters and symptoms [1-4]. Hospital-centered clinical assessments, although essential, may not fully capture the physiological parameters and symptoms affecting quality of life and daily activities. A solution could be the Internet of Medical Things, sometimes referred to as the Internet of Health Care Things, which is making a significant impact on health care. In particular, remote patient monitoring via wearables [5], smartphone apps, and Bluetooth devices has increased dramatically [6]. These devices make it possible to continuously monitor physiological and psychological parameters and symptoms in out-of-hospital settings. Several recent studies have shown the feasibility of remote data collection to assess individuals’ health [2,7-10], but most of these studies have focused on health monitoring at home [11]. However, combining both home-based approaches (eg, pulse oximeters and handheld spirometers) and advanced approaches (eg, smartwatches) can offer a comprehensive multimodal assessment approach [2,3,6-8,11-14]. The COVID-19 pandemic that started in 2019 serves as a prime example of the urgent need for robust remote monitoring capabilities [4,15]. This pandemic forced the implementation of remote monitoring capabilities and helped shape the global strategy of the World Health Organization on digital health from 2020 to 2025 [4]. Furthermore, it forced the world into periods of isolation and lockdown, demonstrating the need for remote monitoring capabilities to aid in case prioritization and timely intervention and ensure continuous high-quality patient care [16]. Access to health care services remotely can reduce the burden of in-person health care services and the economic and environmental costs of hospitalization, transportation, and exposure to in-hospital infectious diseases [17,18]. However, the remote monitoring of respiratory diseases cannot be widely adopted before determining its modes, feasibility, usability, and acceptability to patients.

Respiratory diseases comprise a diverse spectrum of conditions affecting patients of all ages with varied symptoms and prognoses [4]. The increasing incidence of respiratory diseases and high mortality rates are global issues [17,19,20]. In 2017, chronic respiratory diseases affected approximately 544.9 million people worldwide [19,21,22]. The actual costs and long-term outcomes of patients with respiratory diseases are challenging to predict because of the varied disease trajectories that individuals experience [1]. Asthma + Lung United Kingdom reported an estimated 12.7 million people with respiratory diseases in the United Kingdom. Of these patients, 1.2 million were diagnosed with chronic obstructive pulmonary disease (COPD), which is the third leading cause of death worldwide [1], and >150,000 were diagnosed with interstitial lung disease (ILD). According to a recent Asthma + Lung UK report, the United Kingdom spends £11 billion (US $13.4 billion) on respiratory diseases each year, with 29% of that budget allocated to COPD [17,23]. Recent studies have demonstrated the importance of timely identification of exacerbations of COPD [24-26]; therefore, longitudinal measurement of symptoms and physiological parameters has the potential to allow for earlier detection [27]. There is currently an unmet need in the care of patients with respiratory diseases [23,28]. Patients with chronic respiratory diseases and those in the postdischarge period following hospitalization because of COVID-19 are particularly vulnerable, and little is known about the changes in their symptoms and physiological parameters [29,30]. New modalities of remote data collection, such as home-based spirometers, wearables, pulse oximeters, and smartphone apps, may provide the opportunity to improve self-management and offer better, more timely information for clinical assessment. Remote monitoring may help bridge the gap between hospital and home for these patients [14,31,32]. However, questions remain regarding the feasibility and acceptability of remote monitoring of physiology and symptoms for patients with respiratory diseases.

### Objectives

The ultimate goal of remote monitoring is to provide practical health care to people with respiratory diseases, facilitate community-based self-management, support early exacerbation detection, and reduce hospitalization [33,34]. In this prospective cohort study, we sought to gain a better understanding of how well patients with chronic respiratory diseases engage with a remote monitoring system. We evaluated the feasibility, adherence, engagement, retention, acceptability, and usability of remote monitoring of respiratory diseases using commercially available wearables (for heart rate, physical activity, and oxygen saturation [SpO_2_]), spirometry, and questionnaires. We hypothesized that remote monitoring using a finger pulse oximeter, wearables, spirometry, and smartphone apps would be feasible, acceptable, and usable for patients with respiratory diseases, including COPD, ILD, and COVID-19.

## Methods

### Study Design

This is a 3-arm prospective observational cohort study that evaluated the feasibility of remotely monitoring physiological parameters and symptoms via a full-scale comprehensive remote monitoring system (Remote Assessment of Disease and Relapses [RADAR]–base platform) over 6 months [35]. Patients were recruited from the Royal Free Hospital and University College Hospital (United Kingdom). The Remote Assessment of Lung Disease and Impact on Physical and Mental Health detailed protocol has been published recently and can be accessed web-based [35]. The study was registered with International Standard Randomised Controlled Trial Number (16275601). Ethics approval was obtained, and patients provided written informed consent.

### Participant Recruitment and Eligibility

A total of 60 patients were recruited for the study between August 2021 and January 2022. The study population included 33% (20/60) of patients with COPD, 33% (20/60) of patients with ILD, and 33% (20/60) of patients with post–COVID-19 condition recruited from a specialized clinic.

Patients were approached at the Royal Free Hospital and University College Hospital in the United Kingdom. The inclusion criteria for the study were a confirmed diagnosis of COPD, ILD, or COVID-19; previous use of a smartphone; willingness to use the study devices; age of >18 years; and, specifically for the COPD cohort, at least 2 previous exacerbations in the past year. The exclusion criteria were an insufficient understanding of the English language and a lack of physical capability to participate.

### Study Procedure

#### Overview

The study protocol was developed through the joint involvement of both clinicians and patients. The research team screened and explained the study to potential participants 1 week before recruitment. Interested participants were emailed a patient information sheet and the electronic consent form via REDCap (Research Electronic Data Capture; Vanderbilt University). Participants had to read the patient information sheet before signing the electronic consent form. After signing the consent form, an automatic package of baseline questionnaires was triggered. Upon completing the baseline questionnaires, participants received a package of remote monitoring equipment (eg, handheld spirometer, wearable device, finger pulse oximeter, and sometimes a smartphone).

All recruited participants attended enrollment sessions, during which they were taught how to install and use the RADAR questionnaire app, handheld spirometer, and wearable device. All participants received a follow-up call the following day to ensure the appropriate use of the system and were instructed to contact the help desk for support. The study team checked and monitored the data daily. Data that were observed to be missing were investigated, and participants were contacted or notified through reminder messages via the RADAR questionnaire app.

#### Passive Monitoring Outcomes

The Garmin smartwatch (vivoactive 4) collected daily data at different sampling rates. These included recording of SpO_2_ and respiratory rate every minute, heart rate every 15 seconds, sleep tracking daily, stress level and body battery (a proxy for fatigue) every 3 minutes, steps, distance and calories every 15 minutes, and physical activity at dynamic rate based on activities performed by the user.

#### Active Monitoring Outcomes

To determine whether daily self-reported outcome measures predicted significant events, participants received alert notifications via the RADAR questionnaire app to complete mental health questionnaires (fatigue, depression, and anxiety questionnaires). They also received disease-specific questionnaires (COPD assessment test, the living with idiopathic pulmonary fibrosis, and COVID-19 symptoms [structured interview]) and were required to enter their daily finger pulse oximeter reading [1]. Data were collected from the 3 cohorts using the finger pulse oximeter questionnaire, which required users to measure and report their heart rate and SpO_2_ using a finger pulse oximeter. These data were used to evaluate the correlation and assess the accuracy between the readings obtained using the pulse oximeter and those reported by the Garmin device.

#### NuvoAir

Participants were asked to perform 3 unsupervised daily home-based spirometry maneuvers using the NuvoAir platform, which includes a clinician portal, smartphone app, and Bluetooth AirNext spirometer. The participants were supervised in their first session via video call, and the subsequent sessions were unsupervised. They received daily notifications from the RADAR questionnaire app to perform the test.

### Data Collection

This study used the RADAR-base platform, a comprehensive remote monitoring system created to provide high-quality, clinically reliable, real-time, and relevant data for both patients and clinicians [36]. The RADAR-base platform is an open-source mobile health platform used in previous studies such as the RADAR–Central Nervous System and RADAR–Major Depressive Disorder [35,37]. Data were collected using 4 main components. First, REDCap was programmed to automatically send periodic questionnaires, including demographic questionnaires (date of birth, sex, ethnicity, height, weight, and smoking history), medical history, and other health-related questionnaires [35]. Second, a multidimensional questionnaire and speech and vocalization sampling were administered using the RADAR-base active mobile phone app. Third, data were collected from a number of devices: continuous passive monitoring using the Garmin vivoactive 4 and active monitoring outcomes using a daily finger pulse oximeter. Daily lung function measurements were collected using a NuvoAir spirometer in the COPD and ILD cohorts (Table 1). In addition, an innovative approach to boost retention was piloted in this study. Protocol holidays (eg, for patient travel, fatigue, or loss of interest) were offered to patients who wanted to drop out midway through the study, with the option of returning at a chosen date. These were periods in which participants were offered a break from the study (and its protocol) and could specify a time when they would join again. This helped both the participants and the study as it reduced the burden on participants, provided more control, and reduced protocol fatigue, which in turn helped increase retention, compliance, and adherence in the study. Compliance was calculated including and excluding protocol holiday time.

**Table 1:** Comprehensive schedule of assessments, equipment checks, and outcome measures for participants over a 6-month observation period.

Task and frequency	Screening	Consent	Baseline	3 months	6 months	Observation period (6 months)					
						Daily	Continuous	Weekly	Fortnightly	Monthly	End of study
Equipment setup			✓								
Participant identification											
Informed consent		✓									
Deliver equipment			✓								
Verify that apps and data are OK			✓	✓	✓						
Wearable (Garmin)							✓				
Finger pulse oximeter (HR^a^ and SpO_2_^b^)						✓					
Demographics			✓								
Medical history			✓								✓
MRC^c^			✓	✓	✓						
STOP-BANG^d^			✓	✓	✓						
Epworth Sleepiness Scale			✓	✓	✓						
SGRQ^e^			✓	✓	✓						
PSQI^f^			✓	✓	✓					✓	
GAD-7^g^			✓	✓	✓				✓		
PHQ-8^h^									✓		
FSS^i^								✓			
LIPF^j^			✓			✓					
VAS^k^			✓	✓	✓	✓					
KBILD^l^									✓		
COVID-19 symptoms								✓			
PCFS^m^									✓		
Exacerbation diary						✓					
ERS^n^											
CAT^o^ questionnaire						✓		✓			
Check data								✓		✓	
Acceptability					✓						✓
NuvoAir acceptability											✓
Technology assessment measurement fast form				✓	✓						✓
Retrieve equipment											✓

^a^HR: heart rate.

^b^SpO_2_: oxygen saturation.

^c^MRC: Medical Research Council.

^d^STOP-BANG: STOP-Bang screening tool for obstructive sleep apnoea.

^e^SGRQ: St George’s Respiratory Questionnaire.

^f^PSQI: Pittsburgh Sleep Quality Index.

^g^GAD-7: Generalized Anxiety Disorder–7.

^h^PHQ-8: Patient Health Questionnaire–8.

^i^FSS: Fatigue Severity Scale.

^j^LIPF: Living with Idiopathic Pulmonary Fibrosis Questionnaire.

^k^VAS: Visual Analogue Scale.

^l^KBILD: King’s Brief Interstitial Lung Disease Questionnaire.

^m^PCFS: posthospital COVID fatigue scale.

^n^ERS: European Respiratory Society.

^o^CAT: Chronic Obstructive Pulmonary Disease Assessment Test.

### Outcomes

We evaluated feasibility using a range of participant metrics: adherence, engagement, retention, acceptability, and usability. Further details of the outcome measures are provided in the following sections.

#### Adherence

Adherence (or compliance) is a critical factor that measures the extent to which participants comply with or follow the specified protocol. For remote monitoring technologies, compliance typically involves the completion of various tasks and data inputs at predetermined frequencies as outlined in the study protocol. The completion rate at the end of the study provides a valuable indicator of the participants’ actual compliance with the study protocol. Similar to compliance, the completion rate at the end of the study can serve as an important measure of the participants’ overall adherence to the study protocol.

#### Engagement

Engagement in the context of technology adoption refers to the sustained use of and interaction with a system over time [38]. The technology acceptance model identifies several interconnected processes that influence engagement [39]. To assess engagement in our study, we analyzed the completion or availability of data throughout its duration. However, it is important to note that missing data may not always be indicative of participant disengagement as other factors such as technical issues could also contribute. In addition, wearable wear time can serve as an indicator of user engagement and use patterns with the device.

#### Retention

Retention is a crucial metric in mobile health research that provides insights into the duration of participant engagement before attrition. High levels of attrition can pose significant challenges to the feasibility of a study and potentially compromise the validity of its outcomes. Retention can be assessed by calculating the average length of time that participants contribute to data collection before dropping out, which is an essential component of study design and management.

#### Acceptability

Acceptability is considered in this case to be a measure of how well participants evaluated their perceived tolerance of the study protocol and data collection system. This includes (1) the quality, reliability, and usability of the remote monitoring apparatus (eg, wearables, RADAR-base questionnaire app, NuvoAir, and smartphone app quality, reliability, and usability from the participants’ perspective); and (2) acceptability and satisfaction (eg, participants’ satisfaction with and acceptability of the remote monitoring system).

### Analysis

#### Adherence

Adherence was assessed using the final adherence rates for various data types, with slight variations in the methods depending on the data source. In some cases, our study protocol allowed for protocol holidays, which required removing gaps in the data and combining the remaining data to add up to a 180-day study period in total. Adherence rates were visually represented using bar charts with CIs and kernel density estimation plots.

For time-series data, the adherence rate was calculated using the following formula:



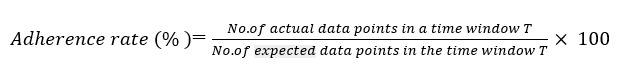



**(1)**

Questionnaire data were evaluated by comparing the expected number of questionnaires based on the protocol with the actual number provided by the patients as a percentage.

For Garmin wearable data, the adherence rate was calculated based on aggregation over hourly windows, and data with negative values (signifying that the metric could not be calculated by Garmin) were marked as missing.

Spirometry adherence rates for the ILD and COPD cohorts were calculated based on the frequency of recordings per week. The American Thoracic Society (ATS) grading system provided with spirometry data was used to assess the quality and usability of the measurements. These analyses enabled us to visualize the optimal protocol frequency for spirometry to limit burden while ensuring useful data.

Adherence was also assessed based on the burden on the patient by grouping the results according to the burden of data collection of different data types. These included (1) *low-burden questionnaire* (light-burden questionnaire tasks such as patient-reported outcome measures [PROMs]), (2) *high-burden questionnaires* (heavy-burden active tasks such as recording finger pulse oximeter values or providing audio recordings), (3) *highest-burden spirometry* (the spirometry task using the provided smart spirometer), and (4) *lowest-burden wearable* (the collection from the Garmin wearable device without active user involvement).

#### Engagement

##### Contiguity

We defined engagement as a measure of the contiguity of data (ie, the extent to which data were collected without gaps). The level of adherence was divided by the number of days to calculate engagement, which provided an indication of how consistently and continuously patients contributed data to the study. These were represented as time-series heat maps to view the overall engagement and grouped according to the burden of data contribution on patients. Hierarchical clustering was performed to understand the patterns of patient engagement.

Engagement with the wearable device was evaluated using the Garmin wearable wear time, which was calculated using the availability of heart rate data. If heart rate data were present, the device was considered to have been worn at those times. This is an estimation and not a perfect approach to ascertain wear time as the device might not calculate heart rate in some instances (eg, when the fit of the device strap is not correct even though it is being worn).

##### Responsiveness

A second measure of engagement looked at the time to respond to prompting by mobile phone notifications [40].

#### Acceptability

At the end of the study, participants were emailed 3 questionnaires: the technology assessment measurement fast form and acceptability and satisfaction questionnaires. Adverse events and safety (eg, reported adverse events and problems encountered during the study) were recorded in the REDCap log.

#### Retention

Retention of patients in the study was evaluated using Cox regression proportional hazard analysis to calculate the time to event, the event being the dropout of a patient. Kaplan-Meier analysis [41] was used to calculate the probabilities of retention at time points from the enrollment dates. Kaplan-Meier plots were grouped based on cohorts, and data types were grouped based on burden and protocol holidays, with group differences assessed using the log rank test.

In total, 2 ways of calculating observation periods and defining an event including or excluding protocol holidays were used. The first one included periods with protocol holidays as missing data. An event date in this case was defined as the last data point for the patient. The second excluded protocol holidays, and the start of the first protocol holiday was considered as the patient’s exit from the study. This was done to simulate retention in case no protocol holiday option was provided in the study (assuming that patients would have otherwise dropped out).

### Ethics Approval

Ethics approval was obtained in London from the Health Research Authority and Health and Care Research Wales (21/WM/0087).

## Results

### Patient Characteristics

**Figure 1:**
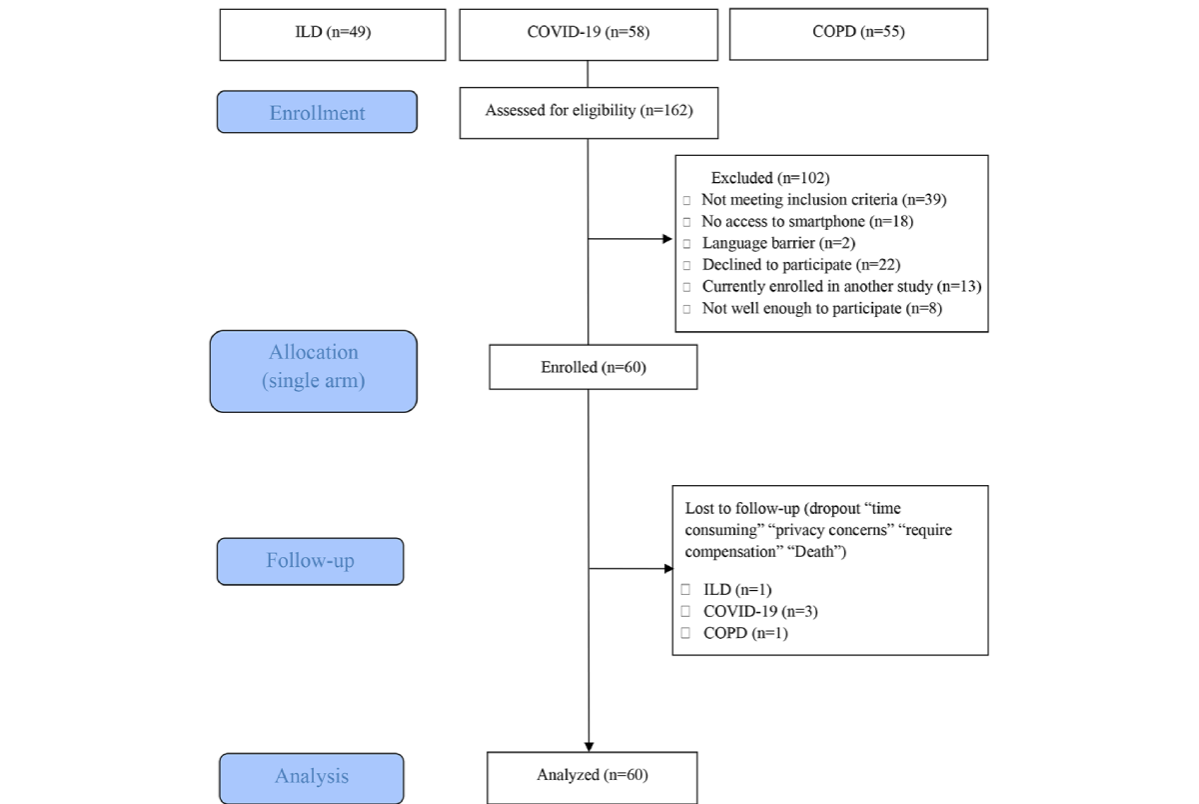
Flowchart of patients screened. COPD: chronic obstructive pulmonary disease; ILD: interstitial lung disease.

A total of 162 patients were assessed for eligibility between July 2021 and November 2022 (Figure 1). In total, 60 recruited patients (ILD: n=20, 33%; COPD: n=20, 33%; post–COVID-19 condition: n=20, 33%) had a retention rate of 55 (92%) across all 3 cohorts at 6 months. A total of 5 patients dropped out of the study: 1 (20%) died during the first week of the study, another (20%) dropped out because they felt unwell, 2 (40%) found the study protocol too time-consuming, and the last patient (20%) dropped out because of privacy concerns.

The 60 patients included in the analysis had a median age of 64 (IQR 36-82) years, with slightly less than half (25/60, 42%) being female. The ILD cohort (11/20, 55%) was more likely to be female than the COPD (9/20, 45%) and COVID-19 (5/20, 25%) cohorts. Other general characteristics are reported in Table 2.

**Table 2:** Baseline characteristics of patients in the Remote Assessment of Lung Disease and Impact on Physical and Mental Health study (N=60)^a^.

Demographic characteristics			Total		ILD^b^ (n=20)		COVID-19 (n=20)		COPD^c^ (n=20)
Gender (women), n (%)			25 (42)		11 (55)		5 (25)		9 (45)
Age (years), median (IQR)			64 (36-85)		60 (36-82)		60 (45-73)		73 (51-79)
BMI (kg/m^2^), mean (SD)			26.37 (6.75)		30.5 (6.5)		28.05 (6.3)		20.55 (7.4)
**Do you live alone, n (%)**									
	No	40 (67)		14 (70)		15 (75)		12 (60)	
	Yes	19 (32)		6 (30)		5 (25)		8 (40)	
**Disability, n (%)**									
	No	32 (53)		13 (65)		13 (65)		6 (30)	
	Yes	28 (47)		7 (35)		7 (35)		14 (70)	
**Issues with fine hand movements, n (%)**									
	No	51 (85)		16 (84)		17 (85)		18 (90)	
	Yes	8 (13)		3 (16)		3 (15)		2 (10)	
**Visual problems, n (%)**									
	No	54 (90)		19 (95)		18 (90)		17 (85)	
	Yes	6 (10)		1 (5)		2 (10)		3 (15)	
**Memory problems, n (%)**									
	No	45 (75)		17 (85)		14 (70)		14 (70)	
	Yes	15 (25)		3 (15)		6 (30)		6 (30)	
**Smoking status, n (%)**									
	Current smoker	3 (5)		2 (10)		0 (0)		1 (5)	
	Ex-smoker	41 (68)		12 (60)		11 (55)		18 (90)	
	Never smoked	16 (27)		6 (30)		9 (45)		1 (5)	
**Use of oxygen, n (%)**									
	No	53 (88)		16 (80)		19 (95)		18 (90)	
	Yes	7 (12)		4 (20)		1 (5)		2 (10)	
**Use of NIV^d^, n (%)**									
	No	52 (87)		17 (85)		19 (95)		16 (80)	
	Yes	8 (13)		3 (15)		1 (5)		4 (20)	

^a^Categorical data are presented as frequencies and percentages, and continuous data are presented as mean and SD or as median and IQR if not normally distributed.

^b^ILD: interstitial lung disease.

^c^COPD: chronic obstructive pulmonary disease.

^d^NIV: noninvasive ventilation.

### Patient Adherence

#### Overview

**Figure 2:**
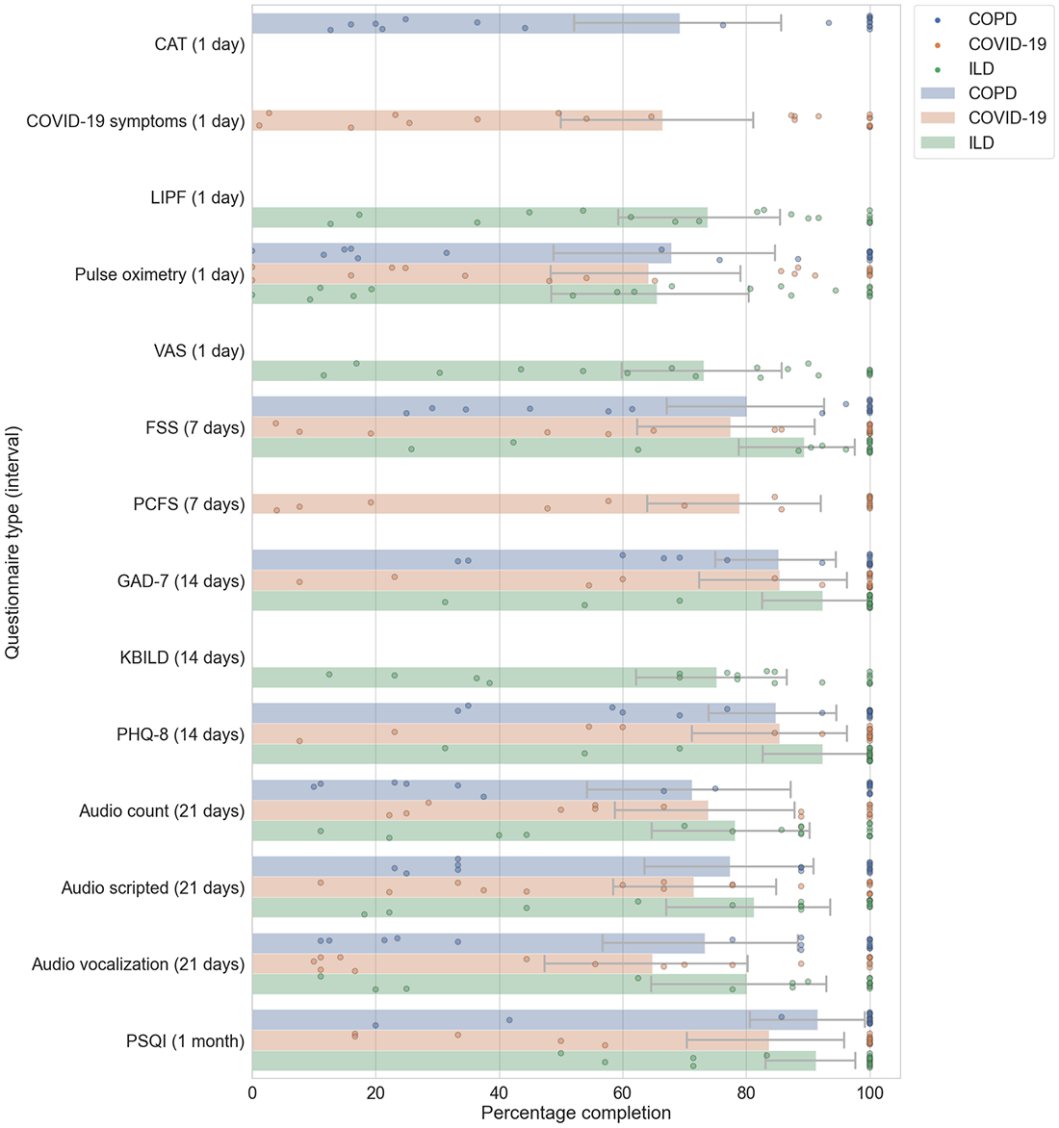
Comparison of adherence rates with 95% CIs across different questionnaire types for the chronic obstructive pulmonary disease (COPD), interstitial lung disease (ILD), and COVID-19 cohorts. CAT: COPD Assessment Test; FSS: Fatigue Severity Scale; GAD-7: Generalized Anxiety Disorder–7; KBILD: King’s Brief Interstitial Lung Disease Questionnaire; LIPF: Living with Idiopathic Pulmonary Fibrosis Questionnaire; PCFS: posthospital COVID fatigue scale; PHQ-8: Patient Health Questionnaire–8; PSQI: Pittsburgh Sleep Quality Index; VAS: Visual Analog Scale.

**Figure 3:**
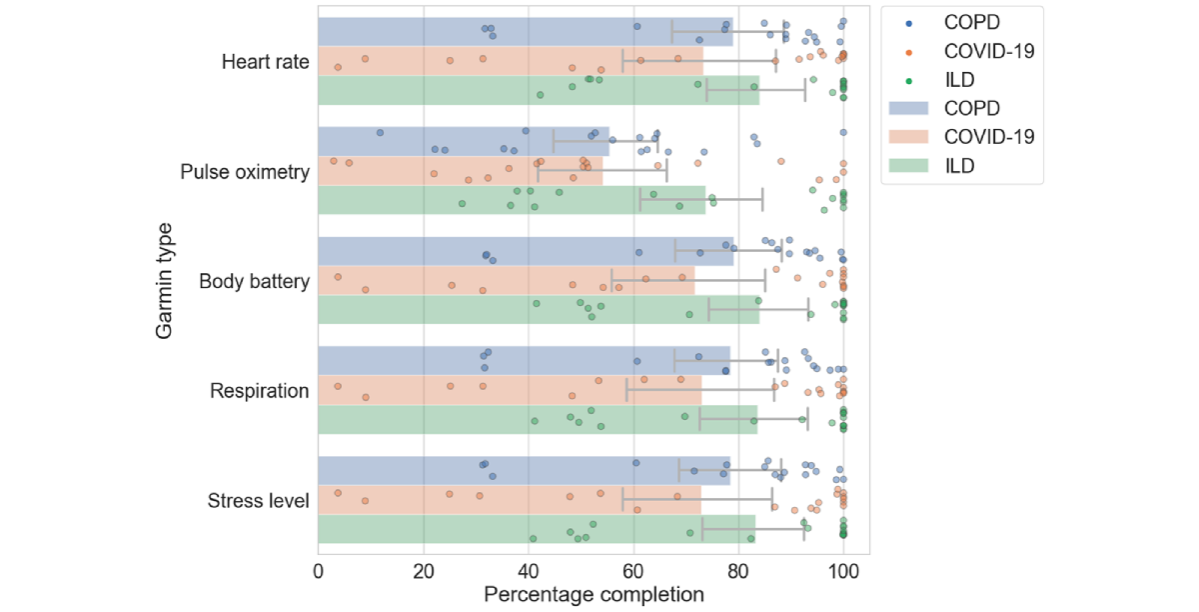
Comparative analysis of percentage of adherence for Garmin wearable data with 95% CIs across the 3 cohorts: chronic obstructive pulmonary disease (COPD), interstitial lung disease (ILD), and COVID-19.

The adherence rate with 95% CIs and kernel density estimations [42] of adherence rate based on questionnaire frequency are shown in Figure 2. When looking at the adherence data grouped by frequency of questionnaires, the questionnaires that were issued with low frequency had the highest adherence, with adherence reducing as the frequency of questionnaires increased in all 3 cohorts. There were some exceptions to this trend, such as the audio questionnaires and spirometry because of the high burden of the task. The *pulse_ox* questionnaire was another high-burden task that required users to measure the pulse rate and SpO_2_ on a finger pulse oximeter device and manually enter the readings into a questionnaire on the mobile app (Table 3). Adherence data from the Garmin wearable device are shown in Figure 3. Overall adherence ranged between 70% and 90% for most data types. The pulse oximeter sensor (SpO_2_) had lower adherence because it was more sensitive to motion artifacts causing issues with the calculation of SpO_2_ during the daytime. The ILD cohort had the best adherence to the wearable device, followed closely by the COPD cohort. The COVID-19 cohort had the lowest adherence.

**Table 3:** Insights into questionnaire adherence within the chronic obstructive pulmonary disease (COPD), interstitial lung disease (ILD), and COVID-19 cohorts.

Cohort and questionnaire			Completion (%)		
			Values, mean (SD)		Values, median
**COPD**					
	Audio count (21 days)	71.2049 (36.546)		100	
	Audio scripted (21 days)	77.3379 (32.1278)		100	
	Audio vocalization (21 days)	73.3145 (36.0394)		88.8889	
	CAT^a^ (1 day)	69.1645 (37.2499)		96.6851	
	FSS^b^ (7 days)	80.0819 (28.845)		100	
	GAD-7^c^ (14 days)	85.1923 (22.9734)		100	
	PHQ-8^d^ (14 days)	84.7293 (23.4477)		100	
	PSQI^e^ (1 month)	91.5212 (22.6389)		100	
	Pulse oximetry (1 day)	67.8662 (39.8041)		94.1989	
**COVID** **-19**					
	Audio count (21 days)	73.8343 (30.145)		88.8889	
	Audio scripted (21 days)	71.466 (30.3564)		77.7778	
	Audio vocalization (21 days)	64.806 (37.2502)		73.8889	
	COVID-19 symptoms (1 day)	66.4124 (36.7143)		87.5691	
	FSS (7 days)	77.4535 (34.001)		100	
	GAD-7 (14 days)	85.3809 (28.2245)		100	
	PCFS^f^ (7 days)	78.8386 (33.3696)		100	
	PHQ-8 (14 days)	85.3809 (28.2245)		100	
	PSQI (1 month)	83.6905 (30.2186)		100	
	Pulse oximetry (1 day)	64.1321 (37.3447)		85.6354	
**ILD^g^**					
	Audio count (21 days)	78.157 (28.841)		88.8889	
	Audio scripted (21 days)	81.2166 (27.6428)		88.8889	
	Audio vocalization (21 days)	80.0817 (31.119)		100	
	FSS (7 days)	89.3691 (21.5894)		100	
	GAD-7 (14 days)	92.333 (19.2738)		100	
	KBILD^h^ (14 days)	75.1474 (27.7182)		83.3333	
	LIPD^i^ (1 day)	73.7338 (28.6467)		82.8729	
	PHQ-8 (14 days)	92.333 (19.2738)		100	
	PSQI (1 month)	91.2281 (16.3047)		100	
	Pulse oximetry (1 day)	65.5348 (36.725)		80.663	
	VAS^j^ (1 day)	73.1141 (29.3646)		82.3204	

^a^CAT: Chronic Obstructive Pulmonary Disease Assessment Test.

^b^FSS: Fatigue Severity Scale.

^c^GAD-7: Generalized Anxiety Disorder–7.

^d^PHQ-8: Patient Health Questionnaire–8.

^e^PSQI: Pittsburgh Sleep Quality Index.

^f^PCFS: posthospital COVID fatigue scale.

^g^ILD: interstitial lung disease.

^h^KBILD: King’s Brief Interstitial Lung Disease Questionnaire.

^i^LIPD: Living with Idiopathic Pulmonary Fibrosis Questionnaire.

^j^VAS: Visual Analogue Scale.

#### Adherence to Home-Based Spirometry

**Figure 4:**
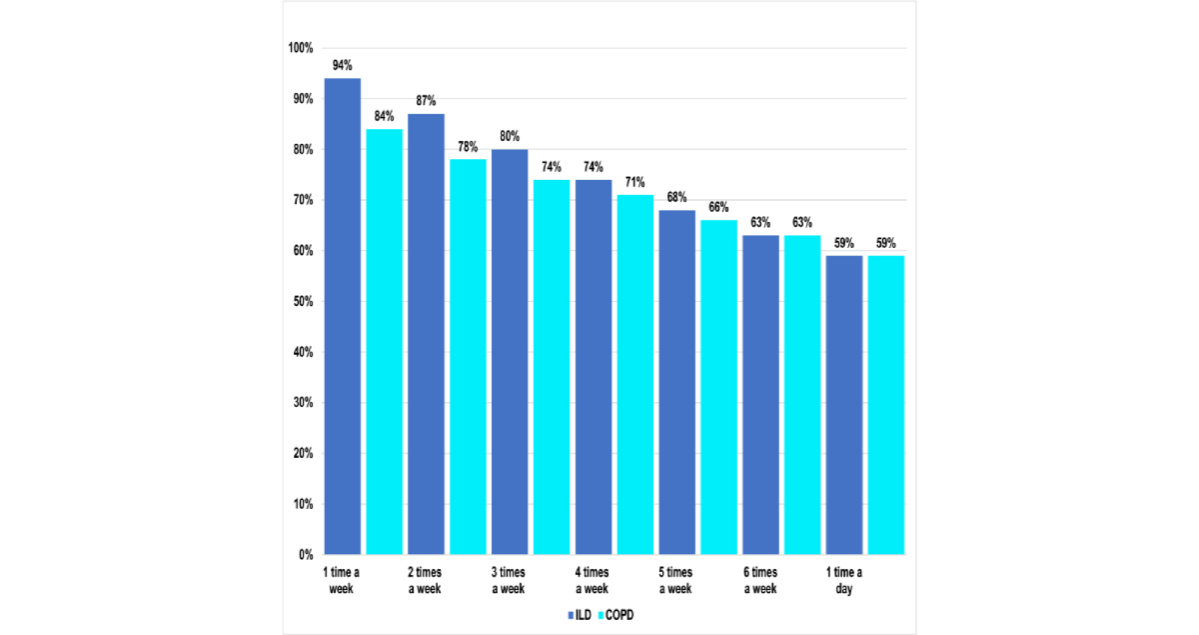
Comparative analysis of adherence rate for home-based spirometry across patients with interstitial lung disease (ILD) and chronic obstructive pulmonary disease (COPD) over different frequencies.

**Figure 5:**
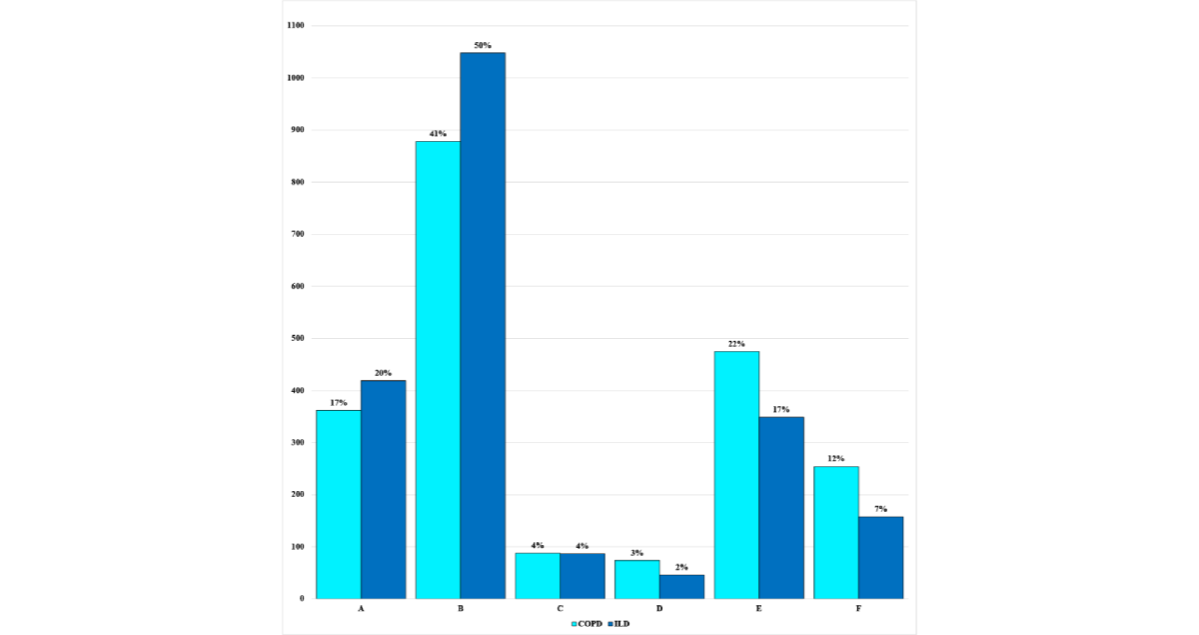
Comparative analysis of home-based spirometry quality according to American Thoracic Society grading criteria across patients with interstitial lung disease (ILD) and chronic obstructive pulmonary disease (COPD). A is the best grade, and F is the worst.

Patients with ILD were found to be more adherent to weekly home-based spirometry than patients with COPD, with adherence rates of 94% and 84%, respectively (Figure 4). A total of 90.3% (3836/4248) of the sessions were of acceptable quality according to ATS grading (grades A-E). Most were classified as grade A (781/4248, 18.39%) or grade B (1926/4248, 45.34%). Others were classified as grades C (175/4248, 4.12%), D (130/4248, 3.06%), and E (824/4248, 19.4%). There were only 9.7% (412/4248) of the sessions that were found to be unacceptable, mostly from the same group of patients (Figure 5).

### Patient Engagement

**Figure 6:**
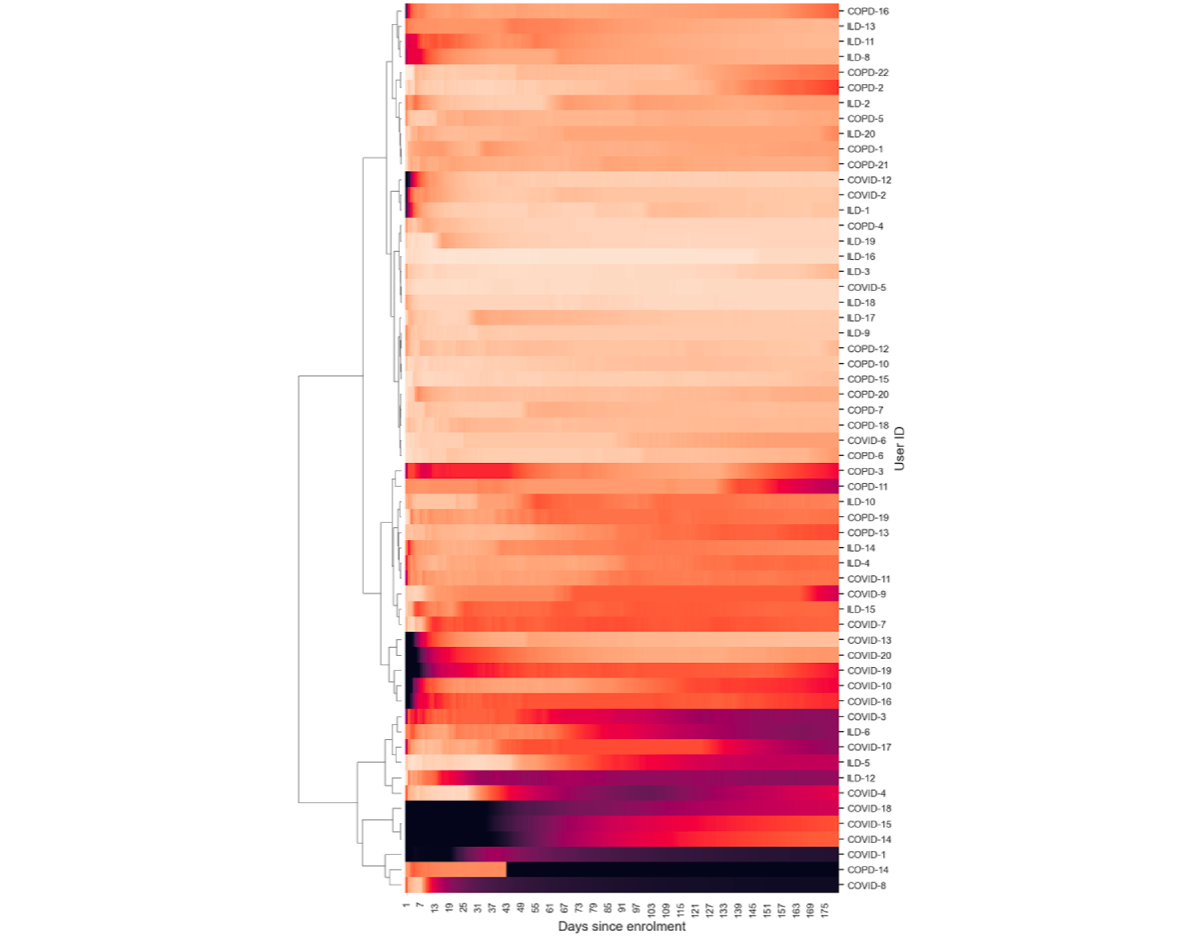
Hierarchical clustering heat maps showing patterns of patient engagement at time points from their enrollment dates for the 3 cohorts for the lowest-burden wearable. The color from dark to light represents completion rates from 0% to 100%. COPD: chronic obstructive pulmonary disease; ILD: interstitial lung disease.

**Figure 7:**
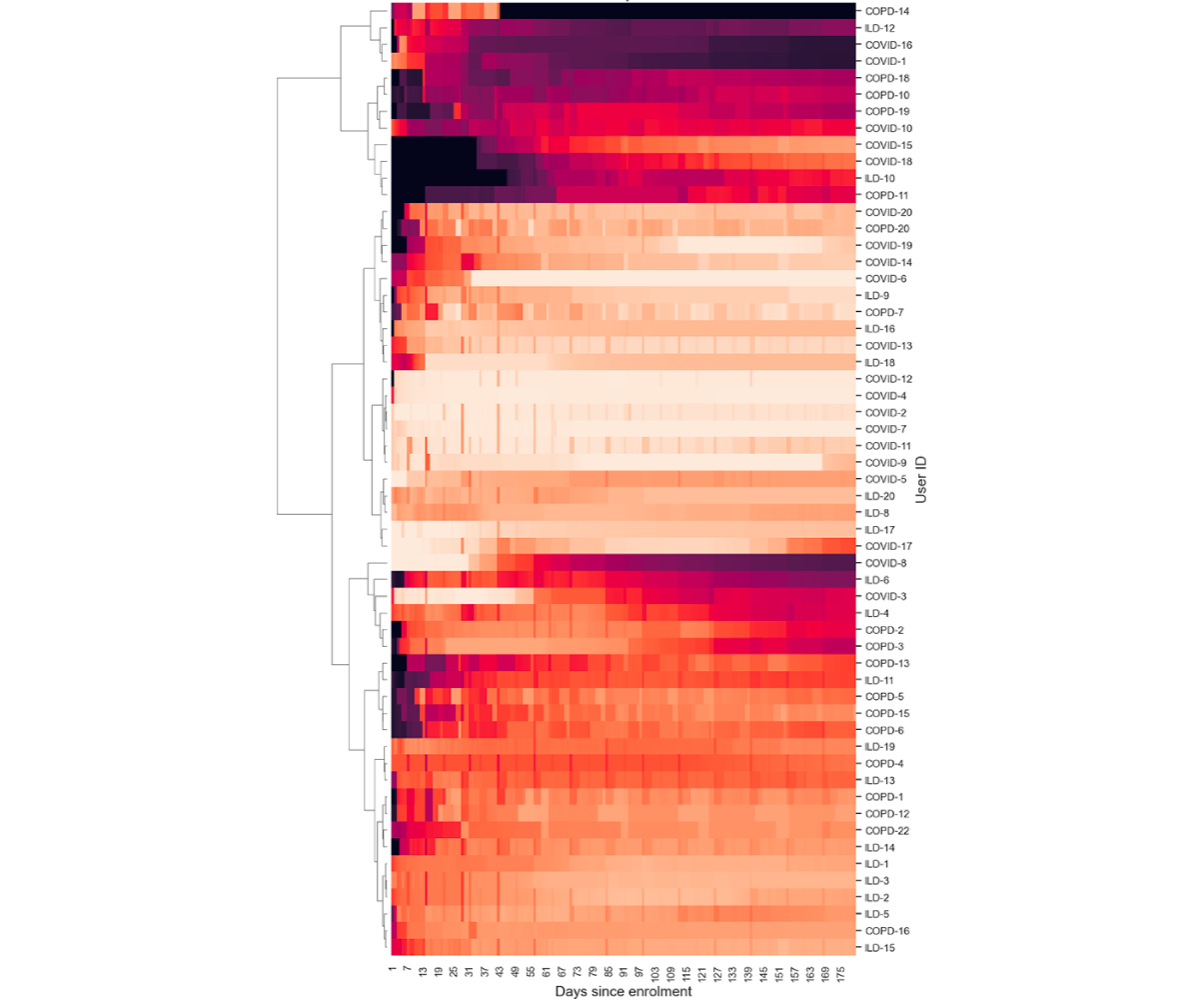
Hierarchical clustering heat maps showing patterns of patient engagement at time points from their enrollment dates for the 3 cohorts for the low-burden wearable. The color from dark to light represents completion rates from 0% to 100%. COPD: chronic obstructive pulmonary disease; ILD: interstitial lung disease.

**Figure 8:**
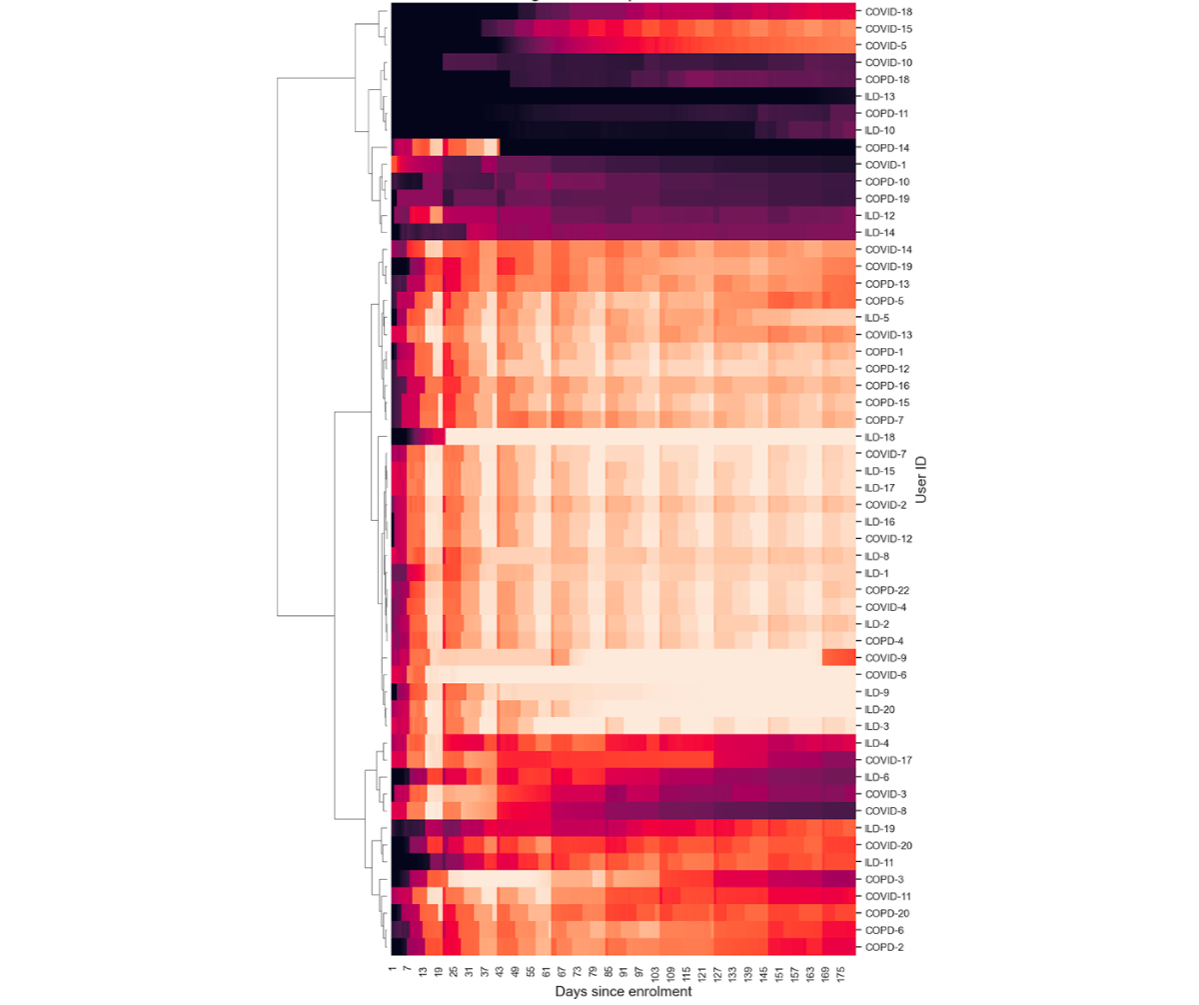
Hierarchical clustering heat maps showing patterns of patient engagement at time points from their enrollment dates for the 3 cohorts for the high-burden questionnaire. The color from dark to light represents completion rates from 0% to 100%. COPD: chronic obstructive pulmonary disease; ILD: interstitial lung disease.

**Figure 9:**
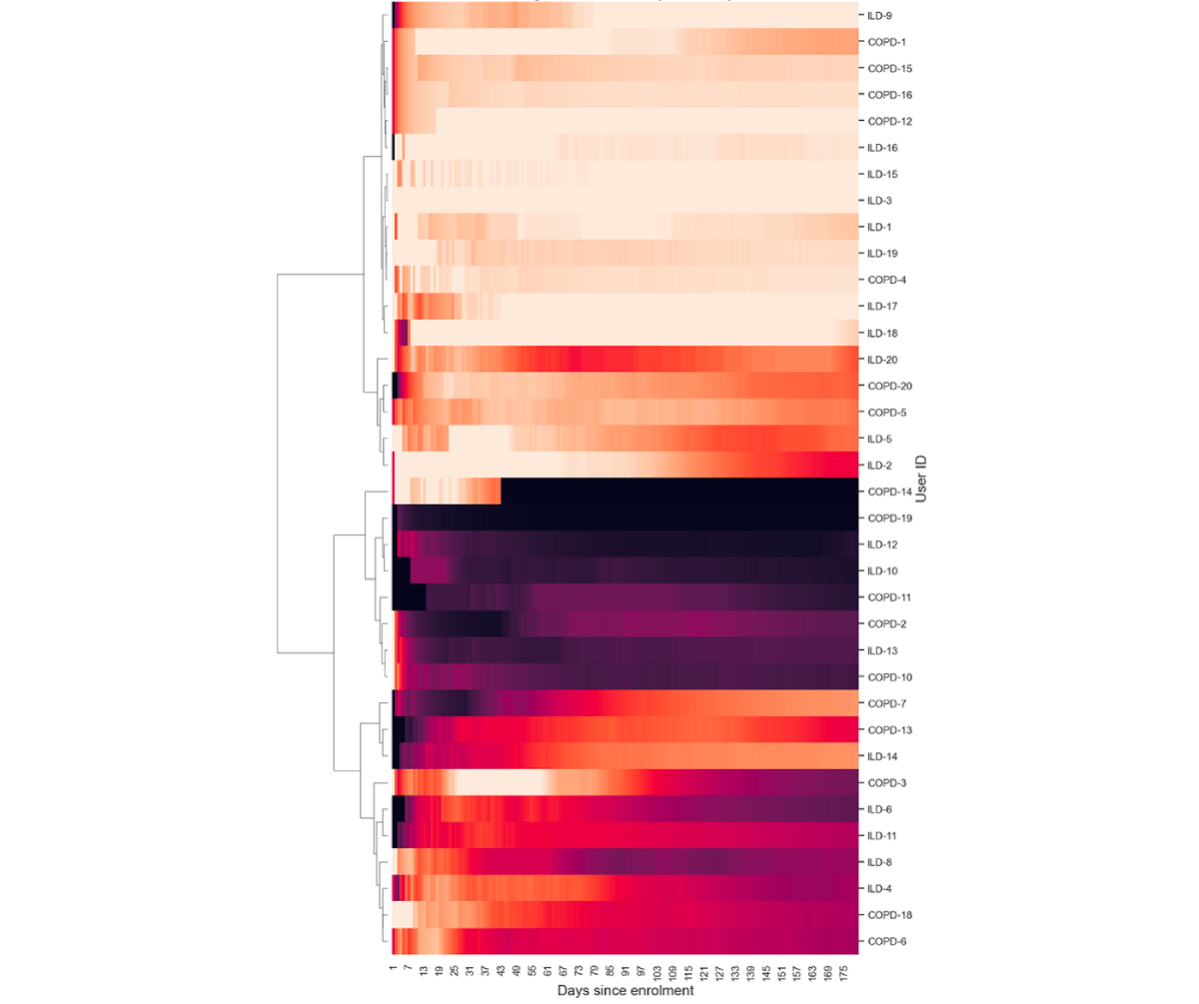
Hierarchical clustering heat maps showing patterns of patient engagement at time points from their enrollment dates for the 3 cohorts for the highest-burden spirometry. COPD: chronic obstructive pulmonary disease; ILD: interstitial lung disease.

Figures 6-9 illustrate patient engagement with wearables, active questionnaires, and spirometry using hierarchical clustering and grouped by the burden of data collection on users. The lighter color represents a higher use rate, whereas a darker color reflects a lower use rate. Each row represents a user, and the y-axis on the right shows the user IDs. Clusters of patients with similar engagement are shown closer to each other on the plots. It can be observed that users from the same cohorts are often clustered together, although there are certain use patterns common among the cohorts. The expected maximum daily use of wearables was approximately 23 hours because of the time needed to charge the device each day (as instructed). The average daily use of wearables varied between patients and ranged from 7.72 to 23.58 hours a day. The average use across all users was 18.66 (SD 4.69) hours daily, 123.91 (SD 33.73) hours (5.16, SD 1.40 days) weekly, and 463.82 (SD 156.70) hours (19.33, SD 6.53 days) monthly.

**Figure 10:**
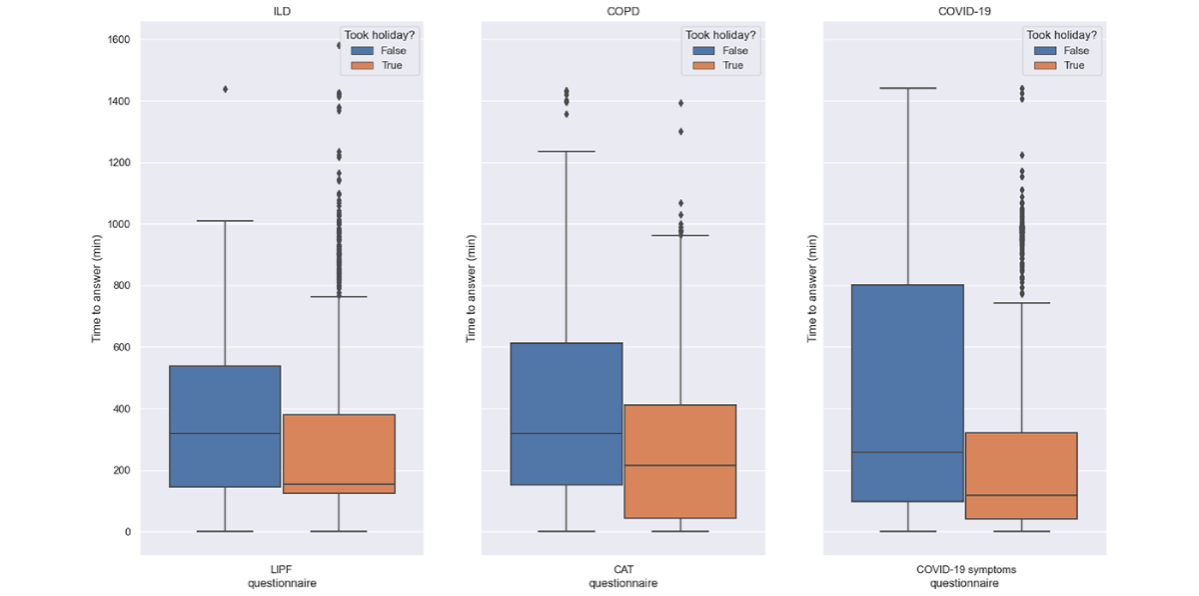
The time to answer a questionnaire, which is defined as the duration between the notification of a questionnaire and its completion (daily study-specific questionnaires). CAT: Chronic Obstructive Pulmonary Disease Assessment Test; COPD: chronic obstructive pulmonary disease; ILD: interstitial lung disease; LIPF: Living with Idiopathic Pulmonary Fibrosis Questionnaire.

**Figure 11:**
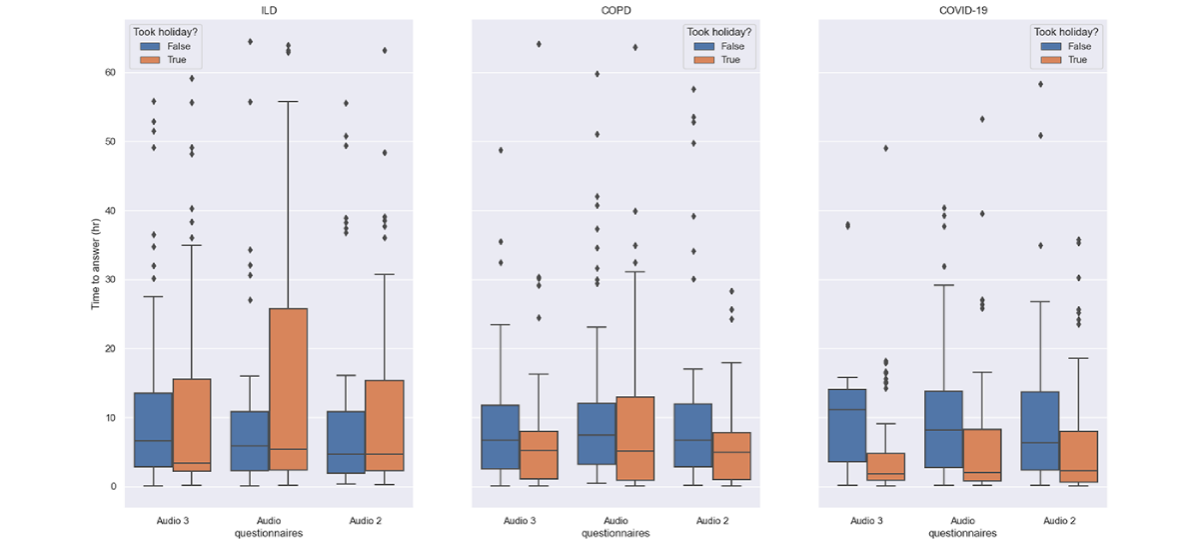
The time to answer a questionnaire, which is defined as the duration between the notification of a questionnaire and its completion (audio questionnaires across the 3 cohorts). COPD: chronic obstructive pulmonary disease; ILD: interstitial lung disease.

To gain a deeper understanding of participant engagement, we examined the time between notification and completion of the questionnaires (Figures 10 and 11). To facilitate the analysis, we grouped participants into 2 categories—those who took protocol holidays and those who did not. We observed that, overall, participants who took holidays had faster response times compared with participants who did not opt for holidays. A similar trend was observed in the COVID-19 cohort, which had faster response times to the questionnaires than the ILD and COPD cohorts.

### Participant Acceptability

**Figure 12:**
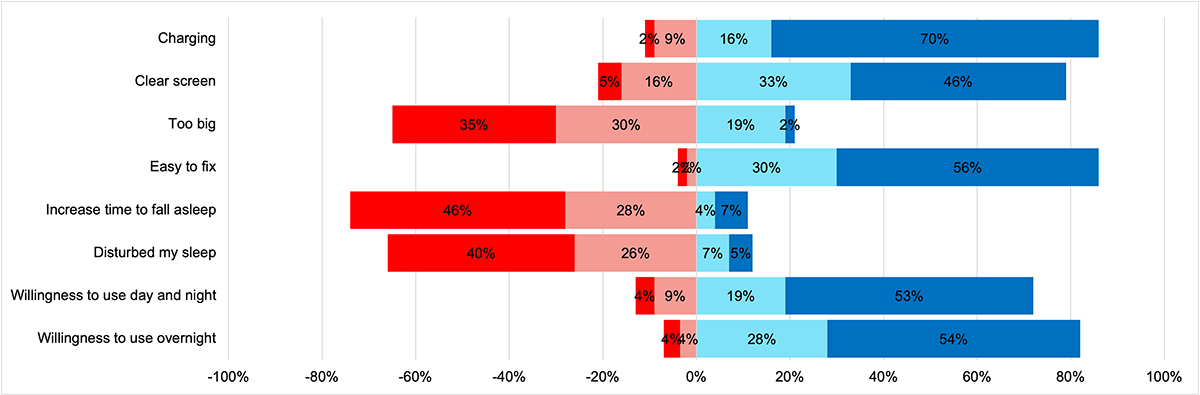
Acceptability of wearable monitoring devices among patients (n=57). Responses are divided into 4 levels: strongly disagree (dark red), disagree (light red), agree (light blue), strongly agree (dark blue).

**Figure 13:**
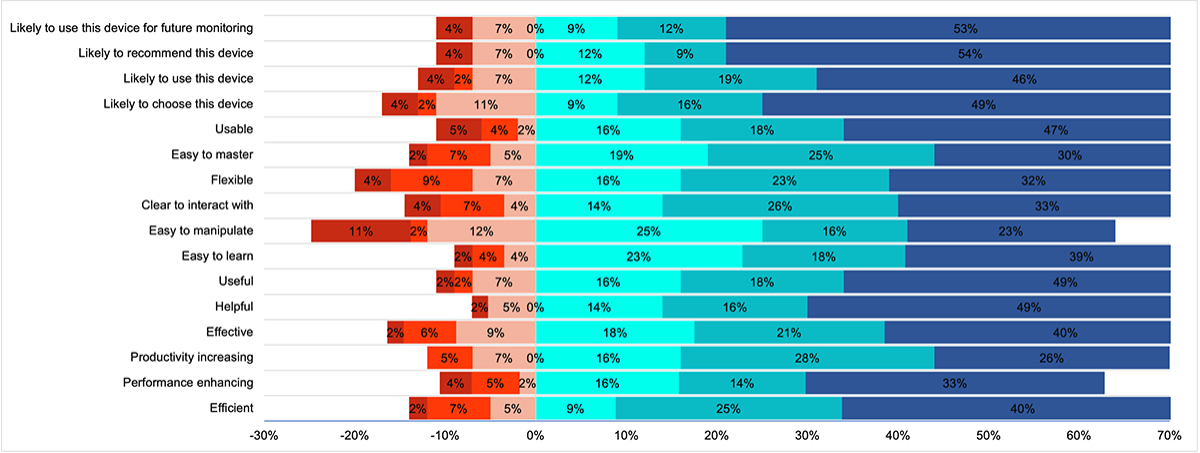
Technology assessment measurement fast form. The responses of patients (n=57) are categorized into 8 levels: strongly disagree (dark red), disagree (light red), somewhat disagree (orangeish red), neither agree nor disagree (very light blue or teal), somewhat agree (lighter blue), agree (medium blue), and strongly agree (dark blue).

**Figure 14:**
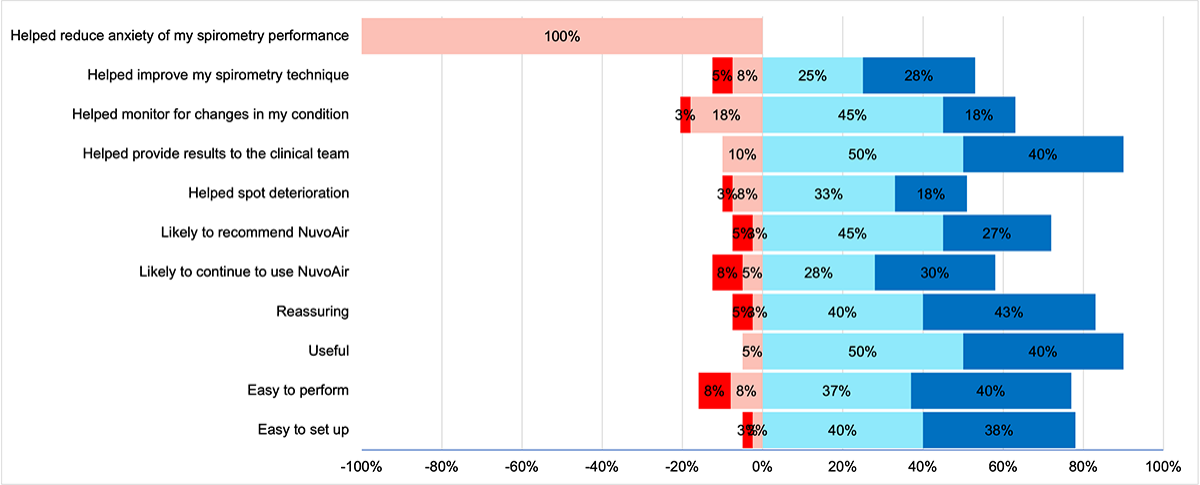
Acceptability of daily home-based spirometry among patients (n=40). Responses are divided into 4 levels: strongly disagree (dark red), disagree (light red), agree (light blue), strongly agree (dark blue).

A high percentage of patients surveyed had a favorable perception of the device’s ease of use and were willing to continue using the device, with (41/57, 72%) stating that they would use it both day and night and (47/57, 82%) showing a willingness to use it overnight (Figure 12). In addition, the technology assessment measurement fast form was used to evaluate patients’ perceptions of acceptance, usefulness, satisfaction, and ease of use. Most patients found the RADAR active app efficient (42/57, 74%), performance enhancing (36/57, 63%), productivity increasing (37/57, 64%), effective (45/57, 79%), helpful (45/57, 79%), and useful (47/57, 83%). Most patients stated that they would likely choose the RADAR app (44/57, 77%), likely use the app (44/57, 77%), and likely use the app for future health monitoring (Figure 13). Finally, most patients (36/40, 90%) found daily home spirometry using the NuvoAir spirometer to be highly acceptable, with high levels of agreement regarding acceptability, usability, and satisfaction (Figure 14).

### Patient Retention

**Figure 15:**
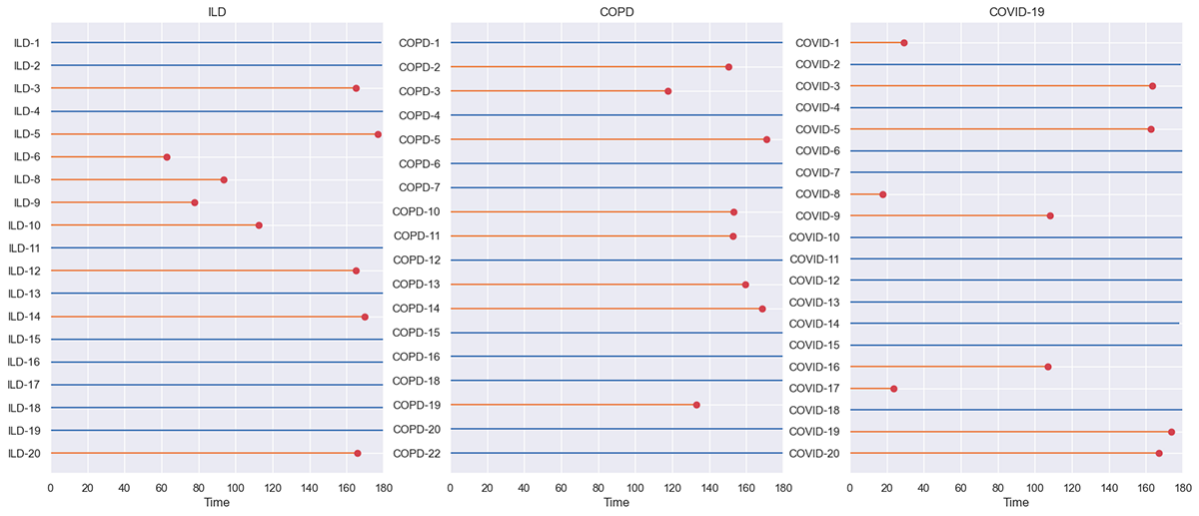
Lifelines of retention for the 3 cohorts. The blue lines represent users who engaged during the full protocol period of 180 days. The orange lines (with markers) represent the users who stopped engaging before the 180-day protocol period. These are based on data averaged across all passive and active sources, including questionnaires and tasks in smartphone apps, Garmin passive data, and spirometry data. COPD: chronic obstructive pulmonary disease; ILD: interstitial lung disease.

**Figure 16:**
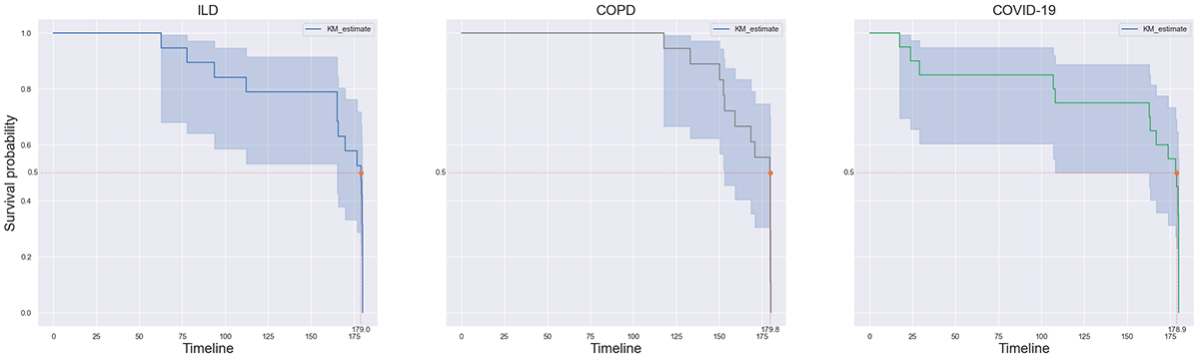
Kaplan-Meier curves for the 3 cohorts with 95% CIs. These are based on data from all passive and active sources, including questionnaires and tasks in smartphone apps, Garmin passive data, and spirometry data. COPD: chronic obstructive pulmonary disease; ILD: interstitial lung disease.

**Figure 17:**
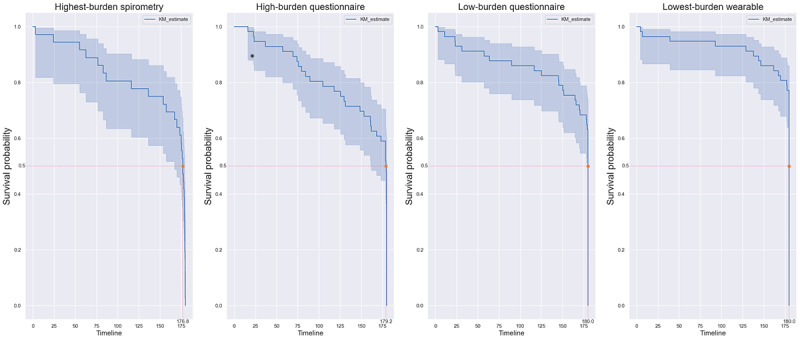
Kaplan-Meier curves based on burden or patient effort required by the data source. Highest-burden spirometry contains questionnaires that require an active action from the user, such as an audio task or measuring finger pulse oximetry using a finger pulse oximeter and reporting it in a questionnaire on the app. Lowest-burden wearable are normal form-type questionnaires that do not require any additional effort. Spirometry can be considered a high-burden task.

A survival analysis was performed to understand user retention in the study. The data were right censored with an observation period of 180 days (the protocol period), and the event was considered as the last data point contributed by the patients in the study. Figure 15 shows the retention of patients in the 3 cohorts. Overall, 54% (31/57) of the patients had a retention of the full 180 days, and 81% (46/57) of the patients had a retention of >150 days (Figure 15). This included patients and data with protocol holidays. Figures 16 and 17 show the survival plots for the 3 cohorts. Even though there was a very good retention at 0.5 survival probability of 179.0, 179.8, 178.9 days in the ILD, COPD, and COVID-19 cohorts, respectively, the COPD cohort had much better retention between 0.8 and 1 survival probability compared with the other cohorts.

### Protocol Holidays

**Figure 18:**
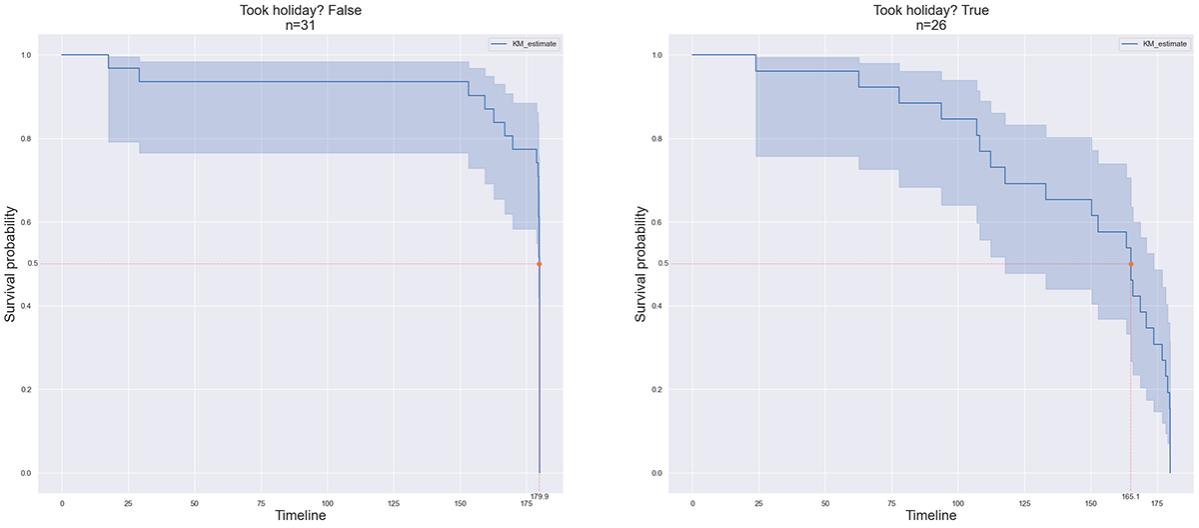
Kaplan-Meier curves based on protocol holidays. The left plot shows retention of patients who did not take any protocol holidays, and the right plot shows retention of patients who opted to take at least one protocol holiday.

**Figure 19:**
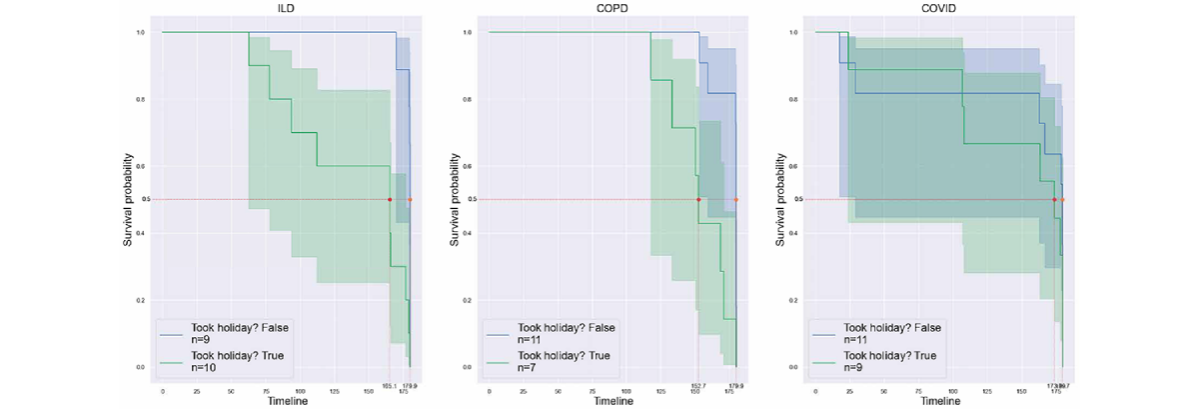
Comparative survival analysis based on protocol holidays per cohort. COPD: chronic obstructive pulmonary disease; ILD: interstitial lung disease.

Protocol holidays were offered to participants who wanted to drop out of the study as a means to boost retention. To compare the differences in retention between participants who required protocol holidays and those who did not, we compared the 2 groups. Figure 18 shows the time-to-last-day Kaplan-Meier survival curves for the 2 groups of patients: those who did not take protocol holidays (group 1) and those who were offered and accepted a protocol holiday (group 2). The retention at 0.5 survival probability was 179.9 and 165.1 days in groups 1 and 2, respectively. To further investigate the differences in retention because of protocol holidays in each cohort, we plotted the retention curves grouped by cohort in Figure 19. The ILD and COPD cohorts showed differences between the groups, but the COVID-19 cohort did not show a discernible difference.

**Figure 20:**
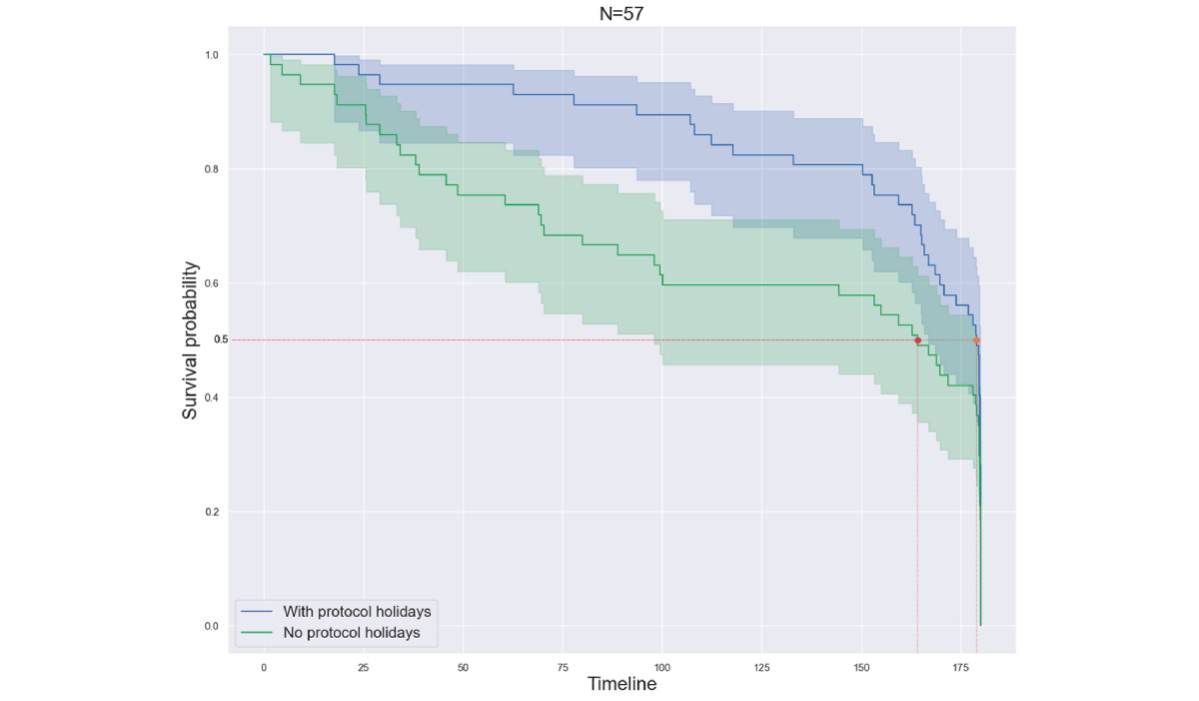
Comparison of retention for all patients based on including or excluding data during protocol holidays for calculating the retention time.

To simulate the effect of data collection without protocol holidays, we used the first protocol holiday as the study exit compared with the full noncontiguous data generated over one or more protocol holidays (Figure 20). In this case, we had 2 groups of data: one inclusive of data after the patient returned to the study from the holiday and another in which we considered the patient as having dropped out at the start of the first protocol holiday, thus marking that as their last day in the study. Note that the patients in both groups were the same; only the selected data differed.

### Reasons for Missing Data

To understand the reasons for adherence and retention, patients were contacted at various points throughout the study, particularly when missing data or low engagement were observed. The patients reported several reasons for the absence of data. Table 4 shows the reasons for these missing data arranged in order of frequency for all 3 cohorts. The most commonly reported reasons for data absence were issues with personal and life circumstances, device and technical challenges, and physical health and accessibility constraints.

**Table 4:** Reasons for missing data ordered by count in all 3 cohorts.

Label	Examples
Personal and life circumstances	Busy with work, school, or family; on vacation; taking a break from the study; privacy concerns about data; not compensated; in rehabilitation
Device and technical challenges	Difficulty using the device, technical malfunctions, problems with the app, inaccurate device readings, compatibility issues with a new phone, synchronization issues
Physical health and accessibility constraints	Unable to perform spirometry due to cough, illness or fatigue; too sick to use the device; hospitalized, participant on life support; device-related sleep issues; skin irritation from wearables
Operational and maintenance issues	Forgot to recharge the device, battery problems, lost charger or device accessories, wrong plug type, accidentally deleting the apps
User experience and motivation	Lost interest, disturbed by device light

^a^RADAR: Remote Assessment of Disease and Relapses.

## Discussion

### Principal Findings

To the best of our knowledge, our study is the first to demonstrate the feasibility and acceptability of the most comprehensive real-time remote monitoring program in patients with a variety of lung diseases using commercially available wearables (for heart rate, activity, and SpO_2_), spirometry, smartphones, and app questionnaires. Despite the COVID-19 pandemic lockdown and the technically complex protocol, patients showed high levels of retention, adherence, and acceptance and reported positive experiences. Patients demonstrated a higher adherence to passive data collection methods than to active data collection methods, which is consistent with what has been previously reported [43]. Adherence was inversely associated with the burden or effort required to complete data collection. Patients were more likely to adhere to weekly active data collection methods than to daily data collection methods. Patients also showed more adherence to the app questionnaire than to the finger pulse oximeter and spirometry data collection, which require extra effort to complete. This can be attributed to the RADAR-base app concept and approach, which were designed to minimize patient burden and prevent inaccuracies in entering manual data. RADAR-base is capable of delivering timely notifications to alert patients as to when to complete tasks. This feature assisted in minimizing the data loss encountered in previous studies [36,44]. Furthermore, the use of real-time data transmission reduces the amount of data loss encountered in remote monitoring [44,45] by providing a direct notification of any significant events or missing data.

### Engagement and Retention

Figures 6-9 show 3 distinct clusters of patient engagement patterns. First, there were patients who exhibited high levels of engagement. Second, there were patients who displayed lower levels of engagement. Finally, there were patients who demonstrated poor adherence across all data types for most of the study duration. In addition, analysis of minor groups or patterns revealed that some patients began the study with poor engagement but improved over time, whereas others started off well but became less engaged as the study progressed. The former was primarily observed in the *high-burden questionnaire* group across all 3 cohorts, which included active audio tasks. This implies that the unique aspects of audio data collection may contribute to this phenomenon and that training effects may lead to improved adherence later in the study.

Retention analysis was performed on data grouped by the level of burden imposed on the patient to complete the data collection. Spirometry and the questionnaires that required active action from the user were the most burdensome, whereas wearable data collection, being passive, was the least burdensome. Figure 17 shows the survival curves based on the burden of data collection. As expected, spirometry had the lowest retention at 0.5 survival probability of 176.8 and 179.2 days, respectively, while also having earlier dropouts. This was followed by *low-burden questionnaire* with a retention of 180 days at 0.5 survival probability. The drop-off in retention in the *lowest-burden wearable* data group was the slowest when looking at survival probabilities between 1 and 0.8.

### Protocol Holidays

We found that implementing “protocol holidays” can boost retention in patients who would otherwise have dropped out of the study. Interestingly, the patients who did opt for protocol holidays had lower retention than those who did not take any protocol holidays, giving insights into the behavioral aspects of various participants in remote monitoring (Figure 18). This can serve as a way to inform recruitment strategies for future studies [46]. Figure 19 displays the differences in retention between the groups per cohort. We observed that the ILD and COPD cohorts showed differences between the groups, but the COVID-19 cohort did not show any discernible differences. Another way in which we analyzed the impact of protocol holidays on retention was by grouping the data collected from patients. One group comprised data after the patient returned to the study from the holiday, and in another group, we considered the patient as having dropped out at the start of the first protocol holiday, thus marking that as their last day in the study. The survival curves for these 2 groups are shown in Figure 20. There were differences between the 2 groups, with the group that included data during protocol holidays showing better retention, particularly between survival probabilities of 1 and 0.5, signifying improvements in retention because of the introduction of protocol holidays.

### Passive Data Collection

Our findings suggest that the use of passive sensing wearables can effectively enable continuous remote monitoring of patients with respiratory diseases. We observed higher adherence, engagement (Figures 2 and 4), and retention rates (Figures 12-14) than previously reported [47,48]. Passive data collection not only ensured minimal data loss but also reduced technical errors. The adherence and retention rates of passive data collection from wearables were notably higher than those of active data collection methods, especially among patients who showed little to no adherence to active data monitoring (as shown in Figures 4 and 14), supporting previously reported findings [40,46]. Garmin wearables provided effortless passive continuous data on heart rate, respiratory rate, activity, and SpO_2_ levels. However, further controlled benchmarking studies would be needed to evaluate the accuracy and usefulness of these data [34]. It also provided quality indications in cases in which the data were not of appropriate quality to calculate derived metrics, such as negative values in stress values, ensuring that high-quality data could be differentiated.

### Active Data Collection

Previous studies using paper diaries have reported challenges with the implementation of PROMs and finger pulse oximeter data in daily care and research [49-51]. Smartphone apps result in better data quality, lower costs, and faster completion times [34,43]. In our study, electronic PROMs were successfully implemented using a smartphone app. The app also facilitated the use of the finger pulse oximeter, which was found to be acceptable and well perceived. This suggests that web-based data collection using a mobile app can facilitate the implementation of electronic PROMs. The burden of active data collection also affected adherence and retention, with low-burden (such as form-based) questionnaires having higher adherence and retention than high-burden questionnaires and audio recordings, as shown in Figures 6-9. Moreover, the COPD and ILD cohorts had higher adherence and retention as they were more hands-on with more contact from the study team, whereas the COVID-19 cohort had the lowest adherence and retention as it was the most hands-off cohort (Figures 15 and 19). In contrast, faster responses to the questionnaires from the COVID-19 cohort were observed, providing behavioral insights compared with the COPD and ILD cohorts. Our findings suggest that considerations such as the app used; interface design; and the number, type, and burden of tasks required should be considered in future studies. Overall, these strategies helped improve patient adherence to the study protocol.

Contrary to previous concerns about the feasibility and reliability of home-based spirometry because of technical issues [44,52], our study demonstrated its feasibility, supporting findings from previous studies [34,44,52-56]. Specifically, the results indicate a higher adherence rate to weekly measurements of 84% in the COPD cohort and 94% in the ILD cohort (Figure 4) compared with those previously reported by Turner et al [57] at 72% in the COPD cohort and by Johannson et al [52] and Noth et al [54] at 90.5% and 86%, respectively, in the ILD cohort. This may be attributed to the use of NuvoAir smart spirometry, which features a user-friendly smartphone app and web-based portal, automatic notifications, periodic notifications through the RADAR-base active application, and the ability of clinicians to send manual reminders to patients. In contrast, previous studies have used alternative data storage methods such as handwritten diary cards and manual downloads for which adherence rates were not reported and quality checks were not applicable [58,59]. Unlike previously reported home-based spirometry reliability [59], the data obtained in this study are considered to be of high quality and reliability because of their validation and quality check process. The device provides feedback on test quality using ATS grading guidelines (shown in Figure 5) and depicts diagrams of inspiration and expiration. However, it is still believed that the study duration, frequency of tests required, data transmission method, and technical issues may have affected the overall quality and adherence rate [55].

### Acceptability

Although the results of our data collection revealed a high level of acceptability and satisfaction with remote monitoring, they also highlighted some areas for improvement. The Garmin wearable was generally well received, particularly regarding ease of use and comfort during wear. However, some participants (38/57, 66%) noted that the lights emitted by the watch disrupted their sleep (Figure 12). The RADAR app was predominantly perceived as useful, efficient, and user-friendly. A high majority of patients (42/57, 74%) indicated that they would continue to use the app and recommend it to others (Figure 13). Home-based spirometry using the NuvoAir spirometer was also favorably accepted (Figure 14). Patients found it reassuring, easy to set up, and simple to perform, consistent with the findings reported previously by Moor et al [60]. However, some patients felt that it was burdensome to perform daily and that it did not alleviate test-related anxiety.

### Missing Data, Technology, and Technical Issues

In our study, we encountered several challenges related to remote monitoring technology and internet connectivity that may have affected patient adherence to the study protocol. These issues included (1) technical difficulties that negatively affected patients’ adherence to the study protocol; (2) difficulty for patients in maintaining motivation to complete the required tasks, particularly when experiencing illness or receiving hospital care; and (3) issues related to the functionality of the apps, including freezing, and problems such as automatic log-out and test errors that required patients to log in repeatedly (Table 4).

To mitigate these challenges in future studies and ensure the success of remote monitoring studies for patients with respiratory diseases, it is crucial to address the reported challenges encountered during our study. It is recommended that strategies such as providing training sessions, having readily available technical support, and offering flexible protocols and task adherence options are implemented to ensure optimal patient experience and adherence. In addition, missing passive wearable data were primarily due to recurrent background synchronization issues with the Garmin devices. These issues are common, and solutions are available on Garmin’s support website, but these are not one-size-fits-all, and a lot of effort, research, and troubleshooting is required of the study management teams. In addition, the need for manual activation of continuous, always-on heart rate and SpO_2_ parameters led to errors and difficulties in activation. In addition, the bright red and green lights emitted by the sensors in the Garmin watch were reported to disrupt patients’ sleep.

### Implications for Future Telemedicine Implementation Efforts

This study was conducted successfully despite the COVID-19 pandemic lockdown. We implemented a simple remote recruitment and onboarding strategy. We also tested modalities with a research team and clinicians and received feedback from patient representatives to better understand the barriers and limitations facing the remote monitoring of patients with respiratory diseases. The low dropout rate shows the feasibility of remote monitoring in clinical research. The reasons for dropout were death, feeling unwell, traveling, being overwhelmed, and the time commitment. The high adherence rate can be attributed to the involvement of patient representatives, simple remote recruitment, strong team communication, monitoring of data streaming, provision of regular calls, and ensuring ease of communication.

The use of a smart finger pulse oximeter that can connect wirelessly to a mobile device and upload the data without extra effort from the patients will further improve the adherence and acceptability of finger pulse oximetry.

### Limitations and Strengths

Our study has several strengths, but it also has some limitations. The main limitation is the small sample size (N=60) and the limited duration of data collection (6 months). This was acceptable for a pilot feasibility study, but a larger sample size and longer duration of data collection would be needed to improve the generalizability of the findings. In addition, we acknowledge that COVID-19 is distinct from chronic conditions. However, this study was to evaluate a system for its feasibility and potential in monitoring a broad spectrum of respiratory diseases. Another limitation is that we used a convenience sampling technique of people willing to take part, which may limit the representation of the general population. A more rigorous sampling technique would be recommended in future studies. Finally, patient recruitment was delayed and complicated by the COVID-19 pandemic, which limited in-clinic spirometry data collection. The study’s strengths stem from a successful data collection exercise, demonstration of daily home-based spirometry, and successful implementation of unique strategies such as protocol holidays and real-time exacerbation state detection. It included a multidimensional investigation of engagement in remote monitoring, shedding light on the technical, statistical, and behavioral aspects of engagement with insights into the impact of burden on adherence, engagement, and retention. It provides a reference point for strategies and policies for future studies to improve adherence, retention, and engagement and how to mitigate common challenges.

### Conclusions

Our study provides valuable evidence supporting the feasibility and acceptance of remote monitoring technologies for patients with respiratory diseases. Patients were more likely to engage with wearables that collected passive data than with technologies that required active input. Flexibility in remote study design, such as allowing for protocol holidays (eg, for patient travel, fatigue, or loss of interest), can also substantially enhance retention and will be more representative of a real-world clinical application of a chronic remote monitoring framework. Our study highlights the value of remote monitoring technologies in clinical research and patient care despite its limitations and challenges. It sets a foundation for future research to improve on these aspects and continue to explore the potential benefits and applicability of remote monitoring, in particular wearables, in broader contexts.

## Abbreviations

ATS: American Thoracic Society
COPD: chronic obstructive pulmonary disease
ILD: interstitial lung disease
PROM: patient-reported outcome measure
RADAR: Remote Assessment of Disease and Relapses
REDCap: Research Electronic Data Capture
SpO2: oxygen saturation

## Data Availability

Data supporting the findings of this study may be requested through the external collaboration process. Data include (1) self-reported questionnaire data; (2) Garmin prospective data, both daily summary and intraday series; and (3) daily spirometry data. These data can be supplied to researchers who provide a methodologically sound proposal. Access to the data is based on collaboration and requires a signed data access agreement. Proposals should be directed to the corresponding author.
